# Frequent avoidable admissions amongst Aboriginal and non-Aboriginal people with chronic conditions in New South Wales, Australia: a historical cohort study

**DOI:** 10.1186/s12913-020-05950-8

**Published:** 2020-11-25

**Authors:** Amanda Jayakody, Christopher Oldmeadow, Mariko Carey, Jamie Bryant, Tiffany Evans, Stephen Ella, John Attia, Simon Towle, Robert Sanson-Fisher

**Affiliations:** 1 Health Behaviour Research Collaborative, School of Medicine and Public Health, Faculty of Health and Medicine, University of Newcastle, Callaghan, NSW 2308 Australia; 2 Priority Research Centre for Health Behaviour, University of Newcastle, Callaghan, NSW Australia; 3 Hunter Medical Research Institute, New Lambton Heights, NSW 2305 Australia; 4 CREDITSS—Clinical Research Design, Information Technology and Statistical Support Unit, Hunter Medical Research Institute, HMRI Building, New Lambton Heights, NSW 2305 Australia; 5 School of Medicine and Public Health, University of Newcastle, Callaghan, NSW 2308 Australia; 6 Nunyara Aboriginal Health Unit, Central Coast Local Health District, Ward Street, Gosford, NSW 2250 Australia; 7 The Cairns Institute, James Cook University, PO Box 6811, Cairns, QLD 4870 Australia

**Keywords:** Aboriginal health, Frequent admissions, Health services research, Data linkage, Chronic disease

## Abstract

**Background:**

Aboriginal and Torres Strait Islander people have high rates of avoidable hospital admissions for chronic conditions, however little is known about the frequency of avoidable admissions for this population. This study examined trends in avoidable admissions among Aboriginal and non-Aboriginal people with chronic conditions in New South Wales (NSW), Australia.

**Methods:**

A historical cohort analysis using de-identified linked administrative data of Aboriginal patients and an equal number of randomly sampled non-Aboriginal patients between 2005/06 to 2013/14. Eligible patients were admitted to a NSW public hospital and who had one or more of the following ambulatory care sensitive chronic conditions as a principal diagnosis: diabetic complications, asthma, angina, hypertension, congestive heart failure and/or chronic obstructive pulmonary disease. The primary outcomes were the number of avoidable admissions for an individual in each financial year, and whether an individual had three or more admissions compared with one to two avoidable admissions in each financial year. Poisson and logistic regression models and a test for differences in yearly trends were used to assess the frequency of avoidable admissions over time, adjusting for sociodemographic variables and restricted to those aged ≤75 years.

**Results:**

Once eligibility criteria had been applied, there were 27,467 avoidable admissions corresponding to 19,025 patients between 2005/06 to 2013/14 (71.2% Aboriginal; 28.8% non-Aboriginal). Aboriginal patients were 15% more likely than non-Aboriginal patients to have a higher number of avoidable admissions per financial year (IRR = 1.15; 95% CI: 1.11, 1.20). Aboriginal patients were almost twice as likely as non-Aboriginal patients to experience three or more avoidable admissions per financial year (OR = 1.90; 95% CI = 1.60, 2.26). There were no significant differences between Aboriginal and non-Aboriginal people in yearly trends for either the number of avoidable admissions, or whether or not an individual experienced three or more avoidable admissions per financial year (*p* = 0.859; 0.860 respectively).

**Conclusion:**

Aboriginal people were significantly more likely to experience frequent avoidable admissions over a nine-year period compared to non-Aboriginal people. These high rates reflect the need for further research into which interventions are able to successfully reduce avoidable admissions among Aboriginal people, and the importance of culturally appropriate community health care.

## Background

The term ‘avoidable admissions’, also known as potentially preventable hospitalisations, refers to hospital admissions for ambulatory care sensitive conditions. Such conditions are considered manageable through timely and effective primary care [1, 2]. Internationally, and in Australia, the concept of avoidable admissions is used as an indicator of health system performance [1, 3, 4]. Chronic conditions which are ambulatory care sensitive include (but are not limited to) chronic obstructive pulmonary disease, diabetic complications and congestive heart failure.

Aboriginal and Torres Strait Islander people (respectfully referred to as Aboriginal people hereinafter) have a higher prevalence of chronic conditions and higher rates of avoidable admissions for chronic conditions compared to non-Aboriginal Australians [3, 5]. Within the Australian state of New South Wales (NSW) avoidable admission rates for chronic diseases are more than three times higher among Aboriginal people compared to non-Aboriginal people [4, 6]. Of particular importance is the fact that these higher rates have remained consistent over the past decade [7].

Among those who experience avoidable hospital admissions, there is a subset of people who are particularly vulnerable due to the frequency of avoidable admissions experienced. Frequent avoidable admissions to hospital are a significant and complex issue facing health services internationally [8–10]. The definition of frequent avoidable admissions varies in the literature, with cut off points at three or four admissions within a 12 months period used [8–11]. However, the most widely reported definition uses three or more admissions within 12 months [8, 11, 12]. Frequent avoidable admissions are associated with a higher risk of an unplanned readmission and are an indication of poor chronic disease management within the community setting [3, 11, 12]. Frequent avoidable admissions are a costly burden on the health system and are a significant cause of bed shortages in hospitals [8, 10]. People who experience frequent avoidable admissions may experience poor quality of life, high out of pocket expenses, psychological distress; and for those most vulnerable patients, frequent admissions can put them at risk of hospital acquired infection [13–15].

The very few research studies that have examined frequent admissions show that Aboriginal people are significantly more likely to experience frequent emergency department presentations and frequent admissions to hospital compared with non-Aboriginal people [9, 16, 17]. A South Australian prevalence study from 2005 to 2011 examining avoidable admissions using linked administrative public hospital record data found that Aboriginal people hospitalised with a chronic condition went on to experience on average 2.6 avoidable admissions in the next 12 months compared to 1.9 avoidable admissions among non-Aboriginal people [18]. Another study examined all inpatient episodes, rather than just avoidable chronic condition admissions, in Northern Territory public hospitals between 2005 and 2013 [9]. Springer and colleagues found that frequent admissions were more common among Aboriginal people (crude odds ratio = 2.50 (95% CI. 2.41–2.59)) compared to non-Aboriginal people, and mostly due to respiratory diseases, injury and poisoning [9]. It is not clear how generalizable the results from these studies are to other Australian states such as NSW which has the largest population of Aboriginal and Torres Strait Islander people in Australia [19]. Relatively little is known about frequent avoidable admissions for Aboriginal people with chronic conditions in NSW.

Examining trends over time in frequent avoidable admissions among Aboriginal people with ambulatory care sensitive chronic conditions has the potential to inform strategies aimed at improving community based chronic disease management. This study examined trends in avoidable admissions among Aboriginal and non-Aboriginal people with ambulatory care sensitive chronic conditions admitted to NSW hospitals between 2005/6 and 2013/14.

## Methods

### Study design

A historical cohort with de-identified linked hospital and administrative data.

### Data sets

The study used data from the NSW Admitted Patient Data Collection (APDC) which was provided by the Centre for Health Record Linkage (CHEREL) [20]. The data collection includes all hospital separations in public and private hospitals in NSW and includes discharges, transfers and deaths. Fact of death was provided by the NSW Registry of Births, Deaths and Marriages (RBDM).

### Study cohort

The study cohort comprised patients who: were aged 18 years and older at the time of index admission; were admitted to a NSW public hospital between 2005/6 and 2013/14; discharged from hospital to the community (reflecting the focus on potentially avoidable admissions which are considered manageable through timely and effective community health care); and had one or more of the following selected ICD-10 defined ambulatory care sensitive (ACS) chronic conditions as a principle diagnosis: diabetic complications, asthma, angina, hypertension, congestive heart failure (CHF) and/or chronic obstructive pulmonary disease (COPD; including Bronchiectasis) (Additional File 1). These chronic conditions were selected as they are highly prevalent among Aboriginal people and an admission to hospital relating to these chronic conditions is considered potentially avoidable through health promotion, preventative measures, or timely access to non-hospital care such as through community health care [3, 4].

### Sampling

The data provided by CHEREL was for the purpose of an overarching analysis project exploring unplanned readmissions [21] and frequent avoidable admissions amongst Aboriginal and non-Aboriginal people (this study). The data provided was restricted to age 18 years and older, and to a selection of chronic conditions (cardiovascular disease, chronic respiratory disease, diabetes and renal disease). The Aboriginal sample included all APDC patients who met this age and chronic disease criteria, had at least one record during the study period, and were documented as Aboriginal and/or Torres Strait Islander on any APDC record. This method was considered the most accurate method available for retrieving Aboriginal status. The level of correct reporting of Aboriginal status in the APDC has been reported to be 90.7% (95% CI 84.6–94.2) [22]. A non-Aboriginal comparison sample was selected by using an equal number of randomly sampled patients who met the age and chronic disease criteria and were not documented as Aboriginal and/or Torres strait Islander on any records. RBDM fact of death pertaining to the sample were included in the final dataset.

### Data preparation

The APDC and RBDM data were provided in a de-identified format by CHEREL. This study’s cohort eligibility criteria (as described above in the Study Cohort section) were applied to the data. Duplicate records were excluded. Periods of care were defined as overlapping episodes of care and sequential transfers were considered in order to define the start and end dates for the period of continuous hospital care. A period of care ended with discharge from hospital. If a patient was discharged and then readmitted the same day, this represented the next period of care. Periods of care in the year of an individual’s death were included in the analysis. Periods of care are referred to as admissions for the remainder of this paper. Two datasets were prepared for analysis: an un-aggregated database of admissions with a defined ACS ICD code (*n* = 31,836) and an aggregated dataset of counts of the number of avoidable admissions for each patient by financial year, and whether they were planned or unplanned admissions (*n* = 22,802).

### Exclusions

Private hospital admissions were excluded from the cohort. It was a priori acknowledged that most private hospital admissions are planned as very few private hospitals have emergency departments, [23] and the majority of hospital admissions for Aboriginal people are in public hospitals (90%) [24]. Planned admissions were excluded from the analysis.

### Analysis variables

For each individual the following outcomes were used: 1) the number of avoidable admissions (defined as an unplanned admission with a principal diagnosis of an ACS chronic condition) for an individual in each financial year (the Australian financial year runs from 1 July to 30 June of the following year); 2) whether or not an individual experienced three or more avoidable admissions in each financial year they were observed over the study period (compared with one to two avoidable admissions). Unplanned admissions were coded as an “emergency status recode” in the APDC.

Patient demographics included in the final dataset were sex, age, Aboriginal status and marital status. The Accessibility/Remoteness Index of Australia (ARIA) and the Index of Relative Socio-economic Disadvantage (IRSD) quintile were calculated. ARIA is an Australian Bureau of Statistics measure of remoteness [25] and the IRSD is a measure of socio-economic status derived from the economic and social conditions within geographic areas [26]. The Charlson Co-morbidity Index (CCI) was calculated [27] which provided a measure of the risk of mortality from comorbidity during the next 12 months. Length of stay was also included.

### Statistical analysis

The denominator for the analysis was all avoidable admissions which met the eligibility criteria. At the admission level (unaggregated data), chi-square and t-tests were used to examine crude associations between Aboriginal status and sociodemographic, disease and admission factors. Then at the patient level (aggregated data), the yearly means of avoidable admissions were calculated by Aboriginal status and financial year. Chi-square tests were then used to examine associations of the proportion of individuals with three or more avoidable admissions compared with one to two avoidable admissions by Aboriginal status and financial year. Multivariable analyses were conducted using the aggregated data. Firstly, a Poisson regression model was used to examine the association of the number of avoidable admissions and Aboriginal status controlling for age, sex, marital status, financial year, IRSD, ARIA and restricted to patients aged ≤75 to account for the younger age structure of the Aboriginal patients. Secondly, a logistic regression model was used to assess the association of three or more avoidable admissions compared with one to two per financial year and Aboriginal status, controlling for age, sex, marital status, financial year, IRSD, ARIA and restricted to patients aged ≤75. To examine any differences in yearly trend between Aboriginal and non-Aboriginal people, an interaction term for Aboriginal status and financial year (as a categorical variable) was included in both final models, followed by a post estimation Wald test of the interaction term. The model was also fit without the interaction term and a post estimation Wald test was used to test the significance of the financial year term. A sensitivity analysis was used to determine any potential differences in results when index admissions ending in death were excluded. The level of type I error was set at 5% for the analysis. Stata software was used for all analyses [28].

### Ethics approval and governance

The Aboriginal Health and Medical Research Council of NSW (AH&MRC) Ethics Committee (1090/15) and the NSW Population & Health Services Research Ethics Committee (HREC/15/CIPHS/18) provided ethical approval for the study. The study complied with ethical guidelines in research, data management and reporting, [29] and core values in research in Aboriginal health: spirit and integrity, cultural continuity, equity, reciprocity, respect, and responsibility [30]. The study advisory committee, which had Aboriginal representation, ensured there was appropriate Aboriginal oversight, and guidance of the study design, methods, analysis and reporting.

## Results

**Fig. 1:**
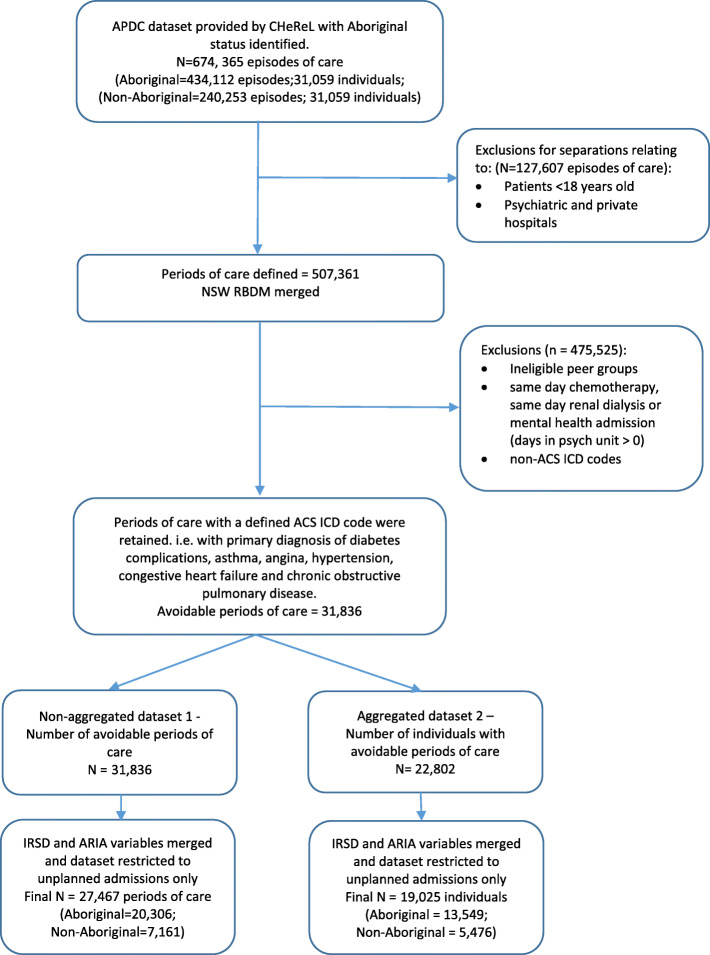
Flow diagram of dataset generation

Once all the eligibility criteria had been applied to the linked dataset (Fig. 1), there was a total of 27,467 avoidable admissions (*n* = 20,306 Aboriginal; *n* = 7161 non-Aboriginal) between the study period 2005/06 to 2013/14.

### Characteristics of people with avoidable admissions

Table 1 describes the characteristics of people with avoidable admissions by Aboriginal status at the admission level between 2005/06 to 2013/14. There were statistically significant differences between Aboriginal and non-Aboriginal patients who experienced avoidable admissions. Aboriginal patients were significantly younger, with an average age of 57 years compared with 70 years in non-Aboriginal people. Aboriginal patients were more likely to be female compared with non-Aboriginal patients, and more likely to be single and divorced. Aboriginal patients had a significantly higher proportion of diabetic complications, asthma and COPD, while non-Aboriginal patients had a significantly higher proportions of angina, hypertension and CHF. Aboriginal patients were also more likely to be socially disadvantaged and live remotely. Lastly, non-Aboriginal patients had a significantly higher median length of stay in hospital compared to Aboriginal patients.

**Table 1:** Characteristics of avoidable admissions by Aboriginal status (admission level) (*n* = 27,467)

		Aboriginal ***% (n)*** (***n*** = 20,306)	Non-Aboriginal ***% (n)*** (***n*** = 7161)	χ2 ***p***-value
**Sex**	% Male	43.9 (8921)	51.5 (3691)	< 0.001
**Age**	Mean (SD)	57.0 (14.9)	69.8 (16.1)	< 0.001
**Marital status**				< 0.001
	Married/de facto	37.1 (7540)	49.7 (3556)	
	Single	29.7 (6023)	11.4 (815)	
	Widowed	15.5 (3148)	26.5 (1898)	
	Divorced/separated	16.0 (3255)	11.4 (813)	
	Not known	1.6 (327)	1.1 (75)	
**Ambulatory care sensitive chronic diseases**	Diabetic complications	18.4 (3746)	13.0 (930)	< 0.001
	Asthma	11.4 (2309)	8.7 (626)	< 0.01
	Angina	17.1 (3466)	20.3 (1452)	< 0.001
	Hypertension	2.4 (493)	3.5 (252)	< 0.001
	CHF	11.2 (2274)	22.5 (1609)	< 0.001
	COPD	39.5 (8018)	32.0 (2292)	< 0.001
**Charlson co-morbidity Index**	0	21.8 (4427)	22.6 (1622)	< 0.001
	1–2	64.9 (13,170)	61.7 (4420)	
	3+	13.3 (2709)	15.6 (1119)	
**Index of relative socio-economic disadvantage (IRSD)**				< 0.001
	1st quintile - most disadvantaged	29.2 (5939)	13.0 (933)	
	2nd quintile	29.9 (6080)	25.4 (1821)	
	3rd quintile	20.7 (4195)	23.0 (1645)	
	4th quintile	16.4 (3329)	21.4 (1535)	
	5th quintile - least disadvantaged	3.8 (763)	17.1 (1227)	
**Accessibility/remoteness index of Australia (ARIA)**				< 0.001
	Highly Accessible	33.5 (6811)	64.9 (4647)	
	Accessible	37.5 (7616)	26.6 (1902)	
	Moderately Accessible	19.2 (3897)	7.3 (525)	
	Remote / Very Remote	9.8 (1982)	1.2 (87)	
**Length of stay**	Median (Interquartile range)	3 (5)	4 (5)	< 0.001

### The number of avoidable admissions by Aboriginal status and financial year

**Fig. 2:**
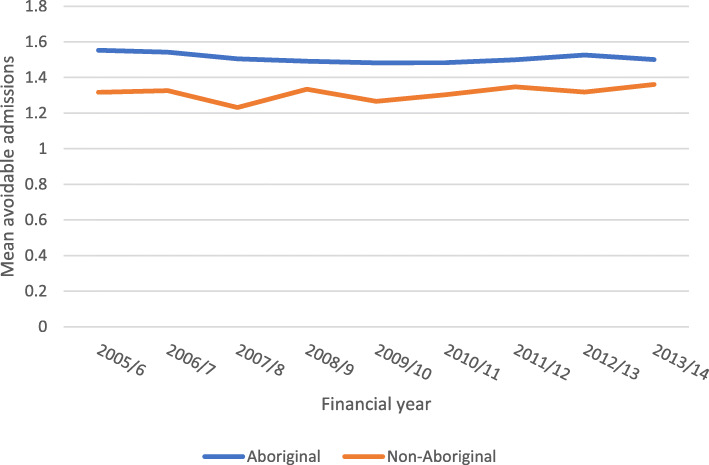
Mean number of avoidable admissions by Aboriginal status and financial year (*n* = 19,025)

At the patient level, the dataset contained a total of 19,025 patients who had experienced avoidable admissions, of which 71.2% were Aboriginal (*n* = 13,549) and 28.8% were non-Aboriginal (*n* = 5476). Averaged across the whole nine-year period, Aboriginal patients had a higher mean of avoidable admissions (Mean = 1.50, Standard deviation = 1.26) compared with non-Aboriginal patients (Mean = 1.30, Standard deviation = 0.84), and this difference remained stable over the study period (Fig. 2).

#### Three or more avoidable admissions per financial year

Table 2 demonstrates the proportion of patients with three or more compared to one to two avoidable admissions each financial year by Aboriginal status. Aboriginal people had a consistently and significantly higher proportion of frequent avoidable admissions over the study period compared with non-Aboriginal people.

**Table 2:** Proportion of patients with three or more compared to one to two avoidable admissions by Aboriginal status and financial year (*n* = 19,025)

Financial year of admission	Aboriginal (***n*** = 13,549) % (n)		Non-Aboriginal (***n*** = 5476) % (n)		***p***-value
	1–2	3+	1–2	3+	
2005/06	88.4 (1102)	11.6 (145)	93.6 (496)	6.4 (34)	0.001
2006/07	89.4 (1139)	10.6 (135)	93.2 (549)	6.8 (40)	0.009
2007/08	89.0 (1234)	11.0 (152)	95.4 (585)	4.6 (28)	< 0.001
2008/09	90.5 (1279)	9.5 (134)	92.6 (525)	7.4 (42)	0.142
2009/10	88.9 (1317)	11.1 (165)	93.8 (515)	6.2 (34)	0.001
2010/11	88.9 (1247)	11.1 (156)	94.3 (525)	5.7 (32)	< 0.001
2011/12	88.8 (1400)	11.2 (177)	93.5 (560)	6.5 (39)	0.001
2012/13	89.5 (1343)	10.5 (157)	93.9 (543)	6.1 (35)	0.002
2013/14	88.9 (1431)	11.1 (179)	92.4 (561)	7.6 (46)	< 0.05

### Regression analyses

At the patient level, unadjusted Poisson regression models were calculated for the number of avoidable admissions for each financial year of the study period (Table 3). Once adjusted for financial year, sex, age, marital status, IRSD and ARIA, Aboriginal patients were 16% more likely than non-Aboriginal patients to have a higher number of avoidable admissions per financial year (IRR = 1.16; 95% CI: 1.13, 1.20). As the age structure of Aboriginal patients was significantly younger, the model was then restricted to patients aged 75 years or less; however Aboriginal patients remained significantly more likely to have more avoidable admissions per financial year (IRR = 1.15; 95% CI: 1.11, 1.20). An interaction term between Aboriginal status and financial year was added to the model which demonstrated no significant difference between Aboriginal and non-Aboriginal people in yearly trends in the number of avoidable admissions each year over the study period (Post estimation Wald test, *p* = 0.860). As the interaction was not significant it was removed from the final model (Table 3). A final post estimation Wald test was conducted on the final model to provide a test of the estimated average yearly trend in both groups however this was not significant (*p* = 0.397).

**Table 3:** Unadjusted and adjusted Poisson regression models for the number of avoidable admissions calculated for each financial year of the study period (2005/06–2013/14) by Aboriginal status and explanatory factors (*n* = 19,025)

	Number of avoidable admissions Incidence rate ratios (IRR) (95% CI)			
	Unadjusted IRR	Adjusted IRR	Adjusted IRR & restricted to < 75 years	***P***-value
**Aboriginal status**				< 0.0001
Non-Aboriginal	ref.	ref.	ref.	
Aboriginal	1.15 (1.12, 1.18)	1.16 (1.13, 1.20)	1.15 (1.11, 1.20)	
**Financial year**				0.397*
2005–06	ref.	ref.	ref.	
2006–07	1.00 (0.94, 1.05)	1.00 (0.94, 1.05)	1.00 (0.93, 1.05)	
2007–08	0.96 (0.91, 1.01)	0.96 (0.91, 1.01)	0.94 (0.89, 1.00)	
2008–09	0.97 (0.92, 1.03)	0.97 (0.92, 1.02)	0.95 (0.90, 1.01)	
2009–10	0.96 (0.91, 1.01)	0.97 (0.92, 1.02)	0.94 (0.88, 0.99)	
2010–11	0.96 (0.91, 1.02)	0.96 (0.91, 1.01)	0.95 (0.89, 1.00)	
2011–12	0.98 (0.93, 1.03)	0.97 (0.92, 1.02)	0.96 (0.91, 1.02)	
2012–13	0.99 (0.94, 1.04)	0.98 (0.93, 1.03)	0.97 (0.92, 1.03)	
2013–14	0.98 (0.93, 1.04)	0.97 (0.93, 1.03)	0.96 (0.90, 1.01)	
**Sex**				
Male	–	ref.	ref.	
Female	–	1.00 (0.97, 1.02)	1.02 (0.99, 1.04)	0.270
**Age**	–	1.00 (1.00, 1.00)	1.00 (1.00, 1.01)	< 0.0001
**Marital status**				
Married	–	ref.	ref.	
Single	–	1.06 (1.03, 1.10)	1.09 (1.05, 1.12)	< 0.0001
Widowed	–	1.04 (1.00, 1.08)	1.09 (1.04, 1.14)	0.001
Divorced/separated	–	1.09 (1.05, 1.13)	1.09 (1.04, 1.13)	< 0.0001
Not known	–	0.98 (0.89, 1.09)	0.99 (0.88, 1.11)	0.833
**Index of relative socio-economic disadvantage (IRSD)**				
1st quintile – most disadvantaged	–	ref.	ref.	
2nd quintile	–	0.98 (0.95, 1.02)	0.99 (0.96, 1.03)	0.787
3rd quintile	–	0.99 (0.95, 1.03)	0.99 (0.95, 1.04)	0.744
4th quintile	–	0.97 (0.93, 1.01)	0.99 (0.94, 1.04)	0.563
5th quintile – least disadvantaged	–	0.92 (0.87, 0.97)	0.88 (0.82, 0.95)	0.001
**Accessibility/remoteness index of Australia (ARIA)**				
Highly Accessible	–	ref.	ref.	
Accessible	–	0.97 (0.94, 1.00)	0.96 (0.93, 0.99)	< 0.05
Moderately Accessible	–	0.98 (0.94, 1.02)	0.95 (0.91, 0.99)	< 0.05
Remote / Very Remote	–	1.02 (0.97, 1.08)	1.00 (0.95, 1.06)	0.924

^a^Post estimation Wald test for financial year term

When looking at whether or not an individual experienced three or more avoidable admissions each financial year, once adjusted for explanatory variables and restricted to ages 75 year or less, Aboriginal patients were almost two times more likely than non-Aboriginal patients to have frequent avoidable admissions per financial year (OR = 1.90; 95% CI = 1.60, 2.26; Table 4). An interaction term between Aboriginal status and financial year demonstrated there were no significant differences between Aboriginal and non-Aboriginal people in yearly trends in the proportion of frequent avoidable admissions over the study period (Post estimation Wald test, *p* = 0.859). As this interaction was not significant, it was removed from the final model (Table 4). There was also no statistically significant average yearly trend in both groups (Post estimation Wald test, p = 0.397).

**Table 4:** Logistic regression model for three or more compared with one to two avoidable admissions: for each financial year of the study period (2005/06–2013/14) by Aboriginal status and explanatory factors (*n* = 19,025)

	≥3 avoidable admissions compared to 1 to 2 per financial year Odds ratios (OR) (95% CI)			
	Unadjusted OR	Adjusted OR	Adjusted OR & restricted to < 75 years	***P***-value
**Aboriginal status**				< 0.0001
Non-Aboriginal	ref.	ref.	ref.	
Aboriginal	1.79 (1.58, 2.03)	1.97 (1.71, 2.27)	1.90 (1.60, 2.26)	
**Financial year**				0.6760^a^
2005–06	ref.	ref.	ref.	
2006–07	0.93 (0.75, 1.16)	0.94 (0.75, 1.17)	0.91 (0.71, 1.16)	
2007–08	0.89 (0.71, 1.10)	0.88 (0.71, 1.10)	0.84 (0.66, 1.07)	
2008–09	0.86 (0.69, 1.08)	0.85 (0.68, 1.06)	0.78 (0.61, 0.99)	
2009–10	0.96 (0.77, 1.18)	0.93 (0.75, 1.16)	0.88 (0.69, 1.11)	
2010–11	0.94 (0.76, 1.17)	0.91 (0.73, 1.13)	0.92 (0.72, 1.17)	
2011–12	0.97 (0.79, 1.20)	0.93 (0.76, 1.15)	0.93 (0.74, 1.17)	
2012–13	0.90 (0.73, 1.11)	0.86 (0.69, 1.07)	0.85 (0.67, 1.08)	
2013–14	0.99 (0.81, 1.23)	0.96 (0.78, 1.18)	0.94 (0.74, 1.18)	
**Sex**				
Male	–	ref.	ref.	
Female	–	0.96 (0.86, 1.06)	1.01 (0.90, 1.13)	0.864
**Age**	–	1.01 (1.01, 1.02)	1.02 (1.01, 1.02)	< 0.0001
**Marital status**				
Married	–	ref.	ref.	
Single	–	1.27 (1.11, 1.45)	1.37 (1.19, 1.59)	< 0.0001
Widowed	–	1.12 (0.96, 1.30)	1.31 (1.09, 1.58)	0.004
Divorced/separated	–	1.33 (1.14, 1.54)	1.34 (1.14, 1.57)	< 0.0001
Not known	–	0.92 (0.59, 1.45)	0.90 (0.54, 1.50)	0.694
**Index of relative socio-economic disadvantage (IRSD)**				
1st quintile – most disadvantaged	–	ref.	ref.	
2nd quintile	–	0.86 (0.75, 0.99)	0.89 (0.76, 1.04)	0.146
3rd quintile	–	1.01 (0.86, 1.18)	1.00 (0.84, 1.20)	0.970
4th quintile	–	0.91 (0.76, 1.10)	1.00 (0.82, 1.23)	0.979
5th quintile – least disadvantaged	–	0.80 (0.63, 1.03)	0.69 (0.50, 0.95)	< 0.05
**Accessibility/remoteness index of Australia (ARIA)**				
Highly Accessible	–	ref.	ref.	
Accessible	–	0.89 (0.78, 1.01)	0.85 (0.73, 0.98)	< 0.05
Moderately Accessible	–	0.98 (0.82, 1.16)	0.89 (0.74, 1.08)	0.245
Remote / Very Remote	–	1.11 (0.90, 1.38)	1.07 (0.85, 1.35)	0.549

^a^ Post estimation Wald test for financial year term

#### Sensitivity analysis

A sensitivity analysis was conducted to examine any potential differences in results when avoidable admissions ending in death were excluded. The regression analyses results were largely similar.

## Discussion

This study has demonstrated that Aboriginal people in NSW are significantly more likely to experience frequent avoidable admissions for ambulatory care sensitive chronic conditions compared with non-Aboriginal people. Aboriginal patients were 15% more likely to have a higher number of avoidable admissions for each financial year over the study period and were almost two times as likely to experience three or more avoidable admissions for each financial year compared to non-Aboriginal people. These findings remained significant after being adjusted for sociodemographic variables.

In our study the rates of both the number of avoidable admissions and whether or not an individual experienced three or more avoidable admissions per financial year remained consistently higher than non-Aboriginal people over the nine-year study period however there were no significant differences in yearly trends between Aboriginal and non-Aboriginal people. This finding demonstrates that Aboriginal people with chronic conditions are at a consistently higher risk of experiencing frequent avoidable admissions compared with non-Aboriginal people. Despite the “Closing the Gap” government strategy to reduce disadvantage among Aboriginal people in health, education and employment being in place since 2008, [31] there is no evidence of the gap being closed in the area of frequent avoidable admissions.

Our findings show that the heightened risk of frequent avoidable admissions is relevant to a small proportion of those Aboriginal people experiencing avoidable admissions. Over the study period an average of 11 % of Aboriginal people experienced three or more avoidable admissions compared to just 6 % in non-Aboriginal people. This is consistent with other research in the area of frequent admissions which reiterates the fact that a small proportion of patients account for a disproportionate share of avoidable admissions [10, 16].

Research in the area of frequent avoidable admissions commonly aims to develop risk profiles or risk prediction tools to help identify those patients most at risk [8, 10, 16]. Our study showed that Aboriginal people experiencing avoidable admissions were more likely to be female, younger, single, have diabetes complications, asthma and COPD, live in moderately accessible to very remote locations, and to be more disadvantaged compared with non-Aboriginal people. Further research in identifying a risk profile for this vulnerable group of people would be helpful in creating appropriate community medical and prevention care.

The high risk of frequent avoidable admissions for Aboriginal people in part reflects the higher rate of chronic conditions in the Aboriginal population which accounts for most of the gap in life expectancy compared with non-Aboriginal people [5]. However it also highlights the health inequities and barriers that remain for Aboriginal people in terms of access to community health services. Cultural and locational factors can impede access to appropriate primary and community health care services for Aboriginal people [32]. National survey data shows that Aboriginal people report difficulties in accessing health services and experience discrimination and services not being culturally appropriate [33]. As our findings demonstrated, compared to non-Aboriginal people, Aboriginal people with avoidable admissions were more likely to live remotely. Aboriginal people who live in remote areas can face practical, logistical and financial barriers which impact on the timeliness and effectiveness of health care [34]. For some Aboriginal people there are also high rates of homelessness, food insecurity, lack of transport, complex comorbidities and alcohol misuse [17, 35]. These underlying risk factors and consequences of social disadvantage have enduring effects and may contribute significantly to the disproportionate burden of frequent avoidable admissions among Aboriginal people.

Our study highlights the need to strengthen services that intervene before a patient needs to be admitted to hospital. Effective management of chronic disease in the primary care setting can delay the progression of disease, improve quality of life, increase life expectancy, and decrease the need to be admitted to hospital [3, 36]. However there is little intervention research in the area of frequent avoidable admissions for Aboriginal people with chronic conditions. A Northern Territory cohort study of a community-led case management program using a culturally competent framework to support frequent attenders aimed to address causes of recurrent emergency department presentations among Aboriginal people with complex social and medical backgrounds. The program was able to significantly improve engagement with primary care and reduce emergency department presentations but not frequent hospital admissions [17]. A retrospective analysis of primary care and inpatient records for Aboriginal patients with diabetes, also in the Northern Territory, found that a timely diabetes care plan was associated with better short-term blood glucose control and fewer diabetes-related admissions [37]. Although such studies provide promising results for reducing frequent avoidable admissions in Aboriginal people, there is still a need for rigorous, well-evaluated and culturally-appropriate interventions to provide robust evidence of effective strategies to help reduce frequent avoidable admissions.

Interestingly, our study found that Aboriginal people in this study had a significantly shorter median length of stay compared with non-Aboriginal people. As discussed in our previous paper examining unplanned readmissions in this same cohort, [21] this finding may indicate that Aboriginal patients with chronic conditions in NSW are not receiving adequate health care or are at higher risk of discharge against medical advice resulting in poorer health outcomes and increased risk of readmission or frequent avoidable admissions.

### Limitations

There are several limitations to this study. Firstly, this study excluded certain ambulatory care sensitive chronic conditions, namely nutritional deficiencies, iron deficiency anaemia and rheumatic heart disease, whose frequent admission outcomes may have influenced the results for our study. Secondly our analysis only included a sample of non-Aboriginal admissions compared to all Aboriginal cases, and it is therefore possible that the non-Aboriginal sample may not be representative of all non-Aboriginal people meeting the study eligibility criteria. Thirdly, the ‘ever identified’ method conducted in our data preparation for identifying Aboriginal patients in the linked data has been found to have some limitations, namely that those with more admissions may have at least one false positive record of Aboriginal status which could potentially increase the frequency for patients reported as Aboriginal [38]. Future analyses could compare the ‘ever identified’ algorithm with a more sophisticated algorithm such as the ‘weight of evidence’ to help determine the amount of bias. Lastly, it is important to keep in mind that not all avoidable admissions may be avoidable. While many admissions could have been prevented through effective chronic disease management in the primary care setting, other admissions may reflect necessary admissions for seriously ill patients [39].

## Conclusion

Over the nine year period from 2005/6 to 2013/14, Aboriginal people in NSW were significantly more likely to experience frequent avoidable admissions compared to non-Aboriginal people. This disproportionate risk remained consistent over the study period. The higher rates of frequent avoidable admissions reflect the higher rate of chronic conditions among Aboriginal people but also the need for further intervention research to establish evidence for effective and culturally appropriate programs which can successfully reduce frequent avoidable admissions among this group.

## Supplementary Information

**Additional file 1.** Selection of ambulatory care sensitive chronic diseases included in eligibility criteria as a principal diagnosis.

## Data Availability

The data used in this study came from the Centre for Health Record Linkage and are available from NSW Ministry of Health. However availability of this data is restricted and not freely available to the public without application to the data custodians, NSW Ministry of Health.

## Abbreviations

ABS: Australian Bureau of Statistics
ACS: Ambulatory care sensitive
APDC: NSW Admitted Patient Data Collection
ARIA: Accessibility/Remoteness Index of Australia
CCI: Charlson Co-morbidity Index
CHEREL: Centre for Health Record Linkage
CHF: Congestive heart failure
CI: Confidence interval
COPD: Chronic obstructive pulmonary disease
IRR: Incidence rate ratio
IRSD: Index of Relative Socio-economic Disadvantage
NSW: New South Wales
OR: Odds ratio
RBDM: NSW Registrar of Births, Deaths and Marriages

## References

[1] Purdey S, Huntley A (2013). Predicting and preventing avoidable hospital admissions: a review. J Royal College Physicians Edinburgh.

[2] Centre for Epidemiology and Evidence: Reporting of Aboriginality in hospital data. Retrieved 04/06/2020 http://www.healthstats.nsw.gov.au/Indicator/dqi_era_apd In*.* NSW Admitted Patient Data and Admitted Patient, Emergency Department Attendance and Deaths Register (SAPHaRI), NSW Ministry of Health; 2020.

[3] Australian Health Ministers’ Advisory Council: Aboriginal and Torres Strait Islander Health Performance Framework 2017 Report. In*.*: AHMAC, Canberra; 2017.

[4] Centre for Epidemiology and Evidence: The health of Aboriginal people of NSW: Report of the Chief Health Officer, 2012. In*.*: Sydney: NSW Ministry of Health; 2012.

[5] Vos T, Barker B, Begg S, Stanley L, Lopez AD (2009). Burden of disease and injury in Aboriginal and Torres Strait islander peoples: the indigenous health gap. Int J Epidemiol.

[6] Harrold TC, Randall DA, Falster MO, Lujic S, Jorm LR (2014). The contribution of geography to disparities in preventable hospitalisations between indigenous and non-indigenous Australians. PLoS One.

[7] Centre for Epidemiology and Evidence: Potentially preventable hospitalisations by category and Aboriginality, Chronic conditions, NSW 2006–07 to 2018–19. Retrieved 14/05/2020 http://www.healthstats.nsw.gov.au/Indicator/bod_acshos/Aboriginality_acshos. In*.* Edited by NSW Combined Admitted Patient Epidemiology Data and ABS population estimates (SAPHaRI) NMoH; 2020.

[8] Low LL, Liu N, Wang S, Thumboo J, Ong ME, Lee KH (2016). Predicting frequent hospital admission risk in Singapore: a retrospective cohort study to investigate the impact of comorbidities, acute illness burden and social determinants of health. BMJ Open.

[9] Springer AM, Condon JR, Li SQ, Guthridge SL (2017). Frequent use of hospital inpatient services during a nine year period: a retrospective cohort study. BMC Health Serv Res.

[10] Raven MC, Doran KM, Kostrowski S, Gillespie CC, Elbel BD (2011). An intervention to improve care and reduce costs for high-risk patients with frequent hospital admissions: a pilot study. BMC Health Serv Res.

[11] Longman JM, M IR, Passey MD, Heathcote KE, Ewald DP, Dunn T, Barclay LM, Morgan GG: Frequent hospital admission of older people with chronic disease: a cross-sectional survey with telephone follow-up and data linkage BMC Health Services Res 2012, 12:373. 10.1186/1472-6963-12-373 PMC3504579 23110342

[12] Kirby SE, Dennis SM, Jayasinghe UW, Harris MF (2010). Patient related factors in frequent readmissions: the influence of condition, access to services and patient choice. BMC Health Serv Res.

[13] Saxena S, George J, Barber J, Fitzpatrick J, Majeed A (2006). Association of population and practice factors with potentially avoidable admission rates for chronic diseases in London: cross sectional analysis. J R Soc Med.

[14] Robert Wood Johnson Foundation (2013). The Revolving Door: A Report on U.S. Hospital Readmissions. The Dartmouth Institute for Health Policy and Clinical Practice.

[15] Calver J, Brameld KJ, Preen DB, Alexia SJ, Boldy DP, McCaul KA (2006). High-cost users of hospital beds in Western Australia: a population-based record linkage study. Med J Aust.

[16] Whyatt D, Yap M, Tenneti R, Pearson G, Vickery A (2017). Hospital use in Aboriginal and non-Aboriginal patients with chronic disease. Emerg Med Australasia.

[17] Quilty S, Wood L, Scrimgeour S, Shannon G, Sherman E, Lake B, Budd R, Lawton P, Moloney M. Addressing Profound Disadvantages to Improve Indigenous Health and Reduce Hospitalisation: A Collaborative Community Program in Remote Northern Territory. Int J Environ Res Public Health. 2019;16(22):4306. 10.3390/ijerph16224306 PMC6888622 31698685

[18] Banham D, Chen T, Karnon J, Brown A, Lynch J (2017). Sociodemographic variations in the amount, duration and cost of potentially preventable hospitalisation for chronic conditions among Aboriginal and non-Aboriginal Australians: a period prevalence study of linked public hospital data. BMJ Open.

[19] Australian Bureau of Statistics: 3238.0.55.001 - Estimates of Aboriginal and Torres Strait Islander Australians, June 2016. In*.* Australian Bureau of Statistics, Canberra; 2016.

[20] Centre for Health Record Linkage: [http://www.cherel.org.au]. Accessed 1 Oct 2018.

[21] Jayakody A, Oldmeadow C, Carey M, Bryant J, Evans T, Ella S, Attia J, Sanson-Fisher R (2018). Unplanned readmission or death after discharge for Aboriginal and non-Aboriginal people with chronic disease in NSW Australia: a retrospective cohort study. BMC Health Serv Res.

[22] Bentley JP, Taylor LK, Brandt PG (2012). Reporting of Aboriginal and Torres Strait islander peoples on the NSW admitted patient data collection: the 2010 data quality survey. N S W Public Health Bull.

[23] NSW Health Private Health Facilities [https://www.health.nsw.gov.au/Hospitals/privatehealth/Pages/list-overnight.aspx]. Accessed 1 Aug 2020.

[24] Australian Institute of Health and Welfare: Admitted patient care 2017–18: Australian hospital statistics. In*.*: Health services series no. 90. Cat. no. HSE 225. Canberra: AIHW; 2019.

[25] Commonwealth Department of Health and Aged Care: Revised Edition: Measuring remoteness: accessibility/remoteness index of Australia (ARIA). Occasional papers: new series Number14. Canberra, Australia. In*.* Occasional papers: new series Number14. Canberra, Australia; 2001.

[26] Australian Bureau of Statistics: IRSD: census of population and housing: socio-economic indexes for areas (SEIFA). In*.* ABS, Australia. Accessed 1 November 2017; 2011.

[27] Charlson ME, Pompei P, Ales KL, MacKenzie CR (1987). A new method of classifying prognostic comorbidity in longitudinal studies: development and validation. J Chronic Dis.

[28] StataCorp: Stata Statistical Software: Release 11. In*.*: College Station, TX: StataCorp LP; 2009.

[29] The National Health and Medical Research Council, The Australian Research Council, Universities Australia: National Statement on Ethical Conduct in Human Research 2007. In*.* Commonwealth of Australia, Canberra; 2018.

[30] National Health and Medical Research Council: Ethical conduct in research with Aboriginal and Torres Strait Islander Peoples and communities: Guidelines for researchers and stakeholders. In*.* Commonwealth of Australia: Canberra; 2018.

[31] Department of the Prime Minister and Cabinet: Closing the Gap Prime Minister's report 2018. . In*.*: Canberra: Department of the Prime Minister and Cabinet; 2018.

[32] Stamp KM, Duckett SJ, Fisher DA (1998). Hospital use for potentially preventable conditions in Aboriginal and Torres Strait islander and other Australian populations. Aust N Z J Public Health.

[33] Australian Bureau of Statistics: National Aboriginal and Torres Strait Islander Health Survey: Australia, 2004–05. In*.* ABS Catalogue no. 4715.0 Canberra: Australian Bureau of Statistics; 2006.

[34] Gracey M (2014). Why closing the Aboriginal health gap is so elusive. Intern Med J.

[35] Randall DA, Lujic S, Havard A, Eades SJ, Jorm L (2018). Multimorbidity among Aboriginal people in New South Wales contributes significantly to their higher mortality. Med J Aust.

[36] Li SQ, Gray NJ, Guthridge SL, Pircher SL (2009). Avoidable hospitalisation in Aboriginal and non-Aboriginal people in the Northern Territory. Med J Aust.

[37] Li SQ, Guthridge S, Lawton P, Burgess P (2019). Does delay in planned diabetes care influence outcomes for Aboriginal Australians? A study of quality in health care. BMC Health Serv Res.

[38] Randall DA, Lujic S, Leyland AH, Jorm LR (2013). Statistical methods to enhance reporting of Aboriginal Australians in routine hospital records using data linkage affect estimates of health disparities. Aust N Z J Public Health.

[39] Longman JM, Passey ME, Ewald DP, Rix E, Morgan GG (2015). Admissions for chronic ambulatory care sensitive conditions - a useful measure of potentially preventable admission?. BMC Health Serv Res.

