# Supplementary material for: Frequent avoidable admissions amongst Aboriginal and non-Aboriginal people with chronic conditions in New South Wales, Australia: a historical cohort study
Source: BMC Health Serv Res. 2020 Nov 25;20:1082. doi: 10.1186/s12913-020-05950-8 (PMC7690010; doi:10.1186/s12913-020-05950-8)
Supplement: Supplementary file 1 — Additional file 1. Selection of ambulatory care sensitive chronic diseases included in eligibility criteria as a principal diagnosis. [file 12913_2020_5950_MOESM1_ESM.docx]

| **Chronic conditions** | **ICD-10-AM codes** |
| --- | --- |
| Diabetes complications | E10.0-E10.8, E11.0-E11.8, E12.0- E12.8, E13.0-E13.8, E14.0-E14.8 |
| Hypertension | I10, I11.9 |
| Congestive heart failure | I11.0, I50, J81 |
| Angina | I20, I24.0, I24.8, I24.9 |
| Chronic obstructive pulmonary disease (including Bronchiectasis) | J41-J44, J47, (J20) |
| Asthma | J45, J46 |

**Additional File 1**: Selection of ambulatory care sensitive chronic diseases included in eligibility criteria as a principal diagnosis
